# Novel AM60-SiO_2_ Nanocomposite Produced via Ultrasound-Assisted Casting; Production and Characterization

**DOI:** 10.3390/ma12233976

**Published:** 2019-11-30

**Authors:** Farzan Barati, Mojtaba Latifi, Ehsan Moayeri far, Mohammad Hossein Mosallanejad, Abdollah Saboori

**Affiliations:** 1 Department of Mechanical Engineering, Islamic Azad University, Hamedan Branch, 6518115743 Hamedan, Iran; f.barati@iauh.ac.ir (F.B.); latifimojtaba18@gmail.com (M.L.); 2 Department of Mechanical Engineering, Faculty of Engineering, University of Isfahan, 8174673441 Isfahan, Iran; e_moayerifar@yahoo.com; 3 Department of Materials Engineering, Isfahan University of Technology, 84156-83111 Isfahan, Iran; 4 Department of Applied Science and Technology, Politecnico di Torino, Corso Duca Degli Abruzzi 24, 10129 Torino, Italy; abdollah.saboori@polito.it

**Keywords:** metal matrix composites (MMCs), casting, mechanical properties, magnesium alloy

## Abstract

There has been growing interest in developing new materials with higher strength-to-weight ratios. Therefore, AM60 magnesium alloy reinforced with SiO_2_ nanoparticles was synthesized using ultrasound-casting method for the first time, in this study. We introduced 1 and 2 wt.% of SiO_2_ nanoparticles into the samples. Introduction of nanoparticles led to the grain size drop in MS2 (AM60 + 2 wt.% SiO_2_) samples. In addition, this increased the hardness of samples from 34.8 Vickers hardness (HV) in M (AM60) to 51.5 HV in MS2, and increased the compressive strength of MS2. Improvement of the mechanical properties can be attributed to a combination of Orowan, Hall–Petch and load-bearing mechanisms. However, ductility of the composites decreased with fracture strains being 0.41, 0.39 and 0.37, respectively, for samples M, MS1 and MS2. Fracture surfaces showed shear fracture in both composite samples with microcracks and a more brittle fracture in MS2.

## 1. Introduction

There has been a growing interest in developing new structural materials with higher strength-to-weight ratios [1,2,3,4,5,6]. As the lightest metal, Mg is about 35% lighter than Al, although both of them have similar melting points and strengths. While Mg has the disadvantage of limited ductility since it is a hexagonal closed-packed (HCP) metal, Al is more ductile given by its face-centered cubic FCC structure. Moreover, Mg has a lower elastic modulus (40–45 GPa) than Al (50 GPa) [7]. Magnesium alloys have been increasingly used in aircraft, automotive industry and leisure equipment, e.g., bicycle frames [1]. However, Mg alloys exhibit a relatively low absolute strength, compared to other structural alloys, especially at high temperatures. The applications of most widely used alloys of magnesium which are based on the Mg–Al system are limited to the temperatures around 120 °C [8].

Various alloying elements such as Ca [2], Nd [3], Si [4], and a combination of them [9,10] have been used to enhance the properties of Mg alloys. These works are consisted of addressing the effects of alloying elements on the microstructure, physical, and mechanical properties of the alloys. However, development of metal matrix composites (MMCs), i.e., a combination of metals with another, often non-metallic, phase to produce a novel material having attractive engineering attributes of its own, have been investigated in recent years [11,12]. Various methods have been used to produce MMCs with unique properties [13,14,15,16,17,18,19,20]. MMCs reinforced with ceramic articulates exhibit high strength and elastic modulus, near-isotropic, as well as high-temperature creep resistant properties [21]. The major disadvantage of MMCs is their relatively high cost of fabrication and the reinforcement materials. Therefore, development of cost-effective processes for producing composite materials is an essential factor for expanding their applications. Diversity of available reinforcing materials and the development of new processing techniques are attracting interest in composite materials [8].

The need for magnesium matrix composites is increasing due to the growing demand for lightweight and high-performance parts, and different research has been carried out to develop new magnesium MMCs. Metallic, ceramic particles and carbon nano tubes (CNTs) were added to magnesium alloys in recent investigations [22,23,24]. In these works, pure magnesium [5] and different Mg alloying systems such as AZ31 [6], AZ91 [25,26], AM50 [22] and Elektron21 [27,28] were used along with reinforcing particles, such as SiC, Al_2_O_3_ and AlN. Among other types of Mg cast alloys, Mg–Al–Mn alloys are used whenever ductility is required [29]. AM60 is an Mg–Al–Mn alloy which, besides good ductility, is featured with suitable strength and thermal stability [30,31,32]. Boruni et al. [33] produced an in-situ magnesium-based cast nanocomposite via addition of 2 wt.% nanoparticles of amorphous SiO_2_ to the AZ91C melt and reported improvements in the mechanical properties by the introduction of the particles, as a result of the secondary phases produced during the casting process. Huang et al. [34] synthesised in situ composite by adding SiO_2_ to the Mg melt and addressed the possible thermodynamic reactions of the ceramic particle with the melt. However, the possible strengthening effects of the unreacted SiO_2_ particles were not addressed in these reports. Strictly speaking, none of the previous reports investigated Mg-based composites where AM60 and SiO_2_ crystalline nanoparticles are used as the metal matrix and the reinforcing particle, respectively, considering that the alteration of alloying system can lead to different behaviour by the composite, as evidenced by the previous works [4,5]. Accordingly, the present study aims to investigate the effect of SiO_2_ crystalline nanoparticles on the microstructure and mechanical properties of AM60 alloy. The composite was manufactured using an ultrasound-assisted casting process and the compressive and hardness tests were carried out to investigate the mechanical properties of the nanocomposite product.

## 2. Materials and Methods

### 2.1. Casting Process

The AM60 magnesium alloy (nominal composition tabulated in Table 1) was prepared using a vacuum resistance furnace (YMVR 1500, Yaran, Tehran, Iran) equipped with a graphite crucible. In order to remove the contamination inside the surface pores of the crucible, the graphite crucible was heated up to 1000 °C and cooled down in a furnace prior to the casting procedure. The crucible was then alkaline washed by NaOH before moving to the furnace chamber. The chamber was vacuumed up to 10^−5^ Torr using a rotary pump (RV, Edwards, CA, United States). Then, the melt was super-heated to 700 °C. After that, the alloy was cast in a carbon steel die with cooling rate of 85 °C sec^−1^ to achieve a billet with 52 × 8 mm^2^.

**Figure 1:**
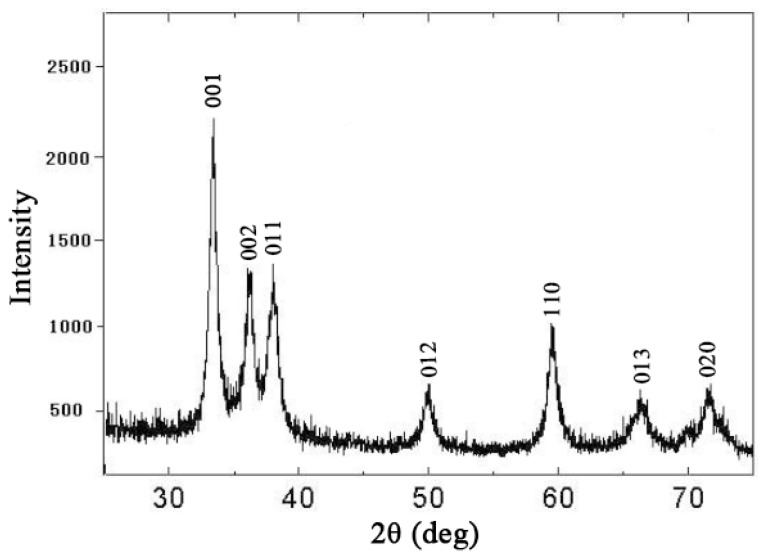
X-ray diffraction (XRD) pattern of the crystalline SiO_2_ nanoparticles used in this research.

The addition of nano crystalline SiO_2_ particles (X-ray diffraction (XRD) pattern is shown in Figure 1) was carried out exactly before the casting process in the carbon steel die. In order to disperse the nanoparticles homogenously, ultrasonication was applied for 5 min at 0.3 kW. On the other hand, the die was designed to allow the fast solidification of the composite melt so that the particles did not have the chance of sedimentation. To investigate the correlation between the reinforcement content and the strengthening efficiency of the nanoparticles, two different composite samples were produced. The composite samples were named MS1 and MS2, where M stands for the Magnesium alloys and S indicates the addition of SiO_2_ nanoparticles with weight percentage being determined by the figure coming at the end.

### 2.2. Characterization

A scanning electron microscope (SEM, MIRA3TESCAN-XMU, TESCAN, Kohoutovice, Czech Republic) equipped with energy-dispersive X-ray spectroscopy (EDS, Munich, Germany) was employed to investigate the morphology, chemical composition and dispersion of the additives. X-ray diffraction (XRD, Philips X’Pert, Philips, Almelo, Netherland) technique was used to analyse the phase composition of the as-cast materials. The XRD patterns were recorded by powder diffraction using a D8 ADVANCE type (BRUKER-Germany) with a Cu-Kα source (λ = Cu-Kα 0.1542 nm).

The Vickers hardness of M and MS2 samples were measured with a 98 N loading. Compressive tests were carried out for samples M, MS1 and MS2 at a fixed strain rate of 9.25 × 10^−4^ s^−1^, without lubrication, and the stress-strain curves were determined. One test was carried out for each sample, and the length to diameter ratio of the samples was selected 1.5 (diameter 6mm and height 9mm) which was in accordance with the ASTM E9 standard.

## 3. Results and Discussion

### 3.1. Morphology and Energy-Dispersive X-ray Spectroscopy (EDS) Analysis

**Figure 2:**
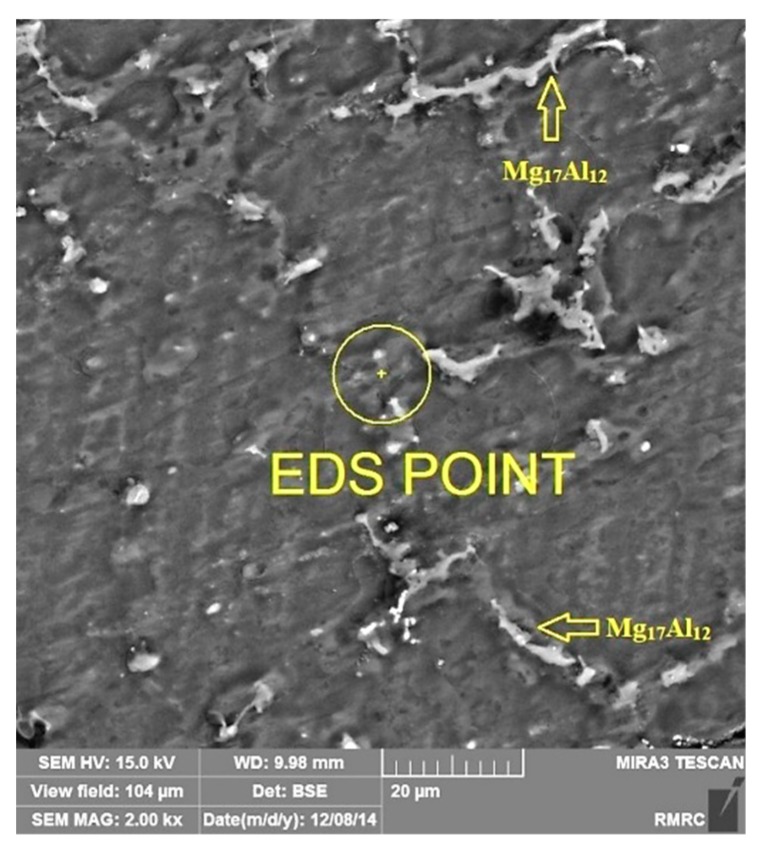
Point energy-dispersive X-ray spectroscopy (EDS) carried out on cross-section of the sample MS2.

**Figure 3:**
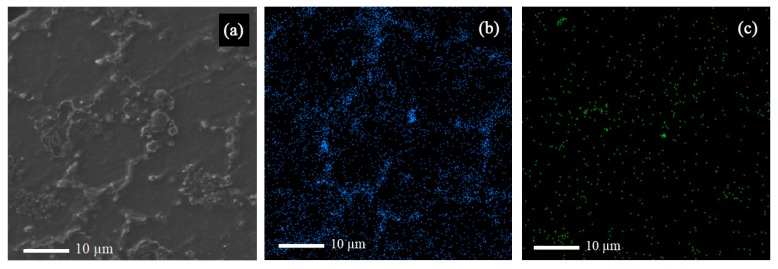
(**a**) Scanning electron microscope (SEM) image of sample MS2 along with the EDS map of (**b**) Al, and (**c**) Si in sample MS2.

**Figure 4:**
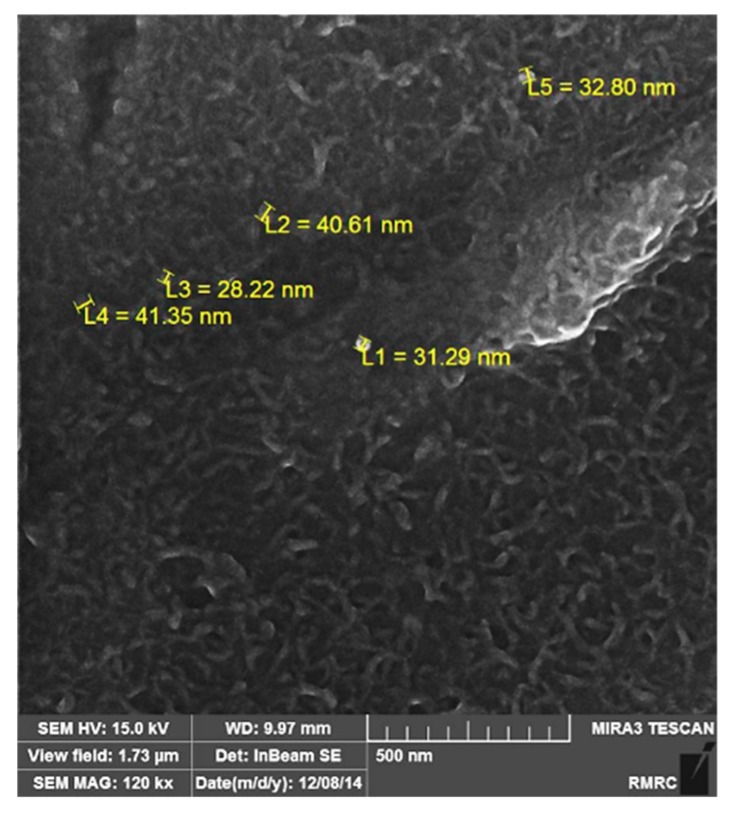
Field-emission scanning electron microscope (FESEM) image of MS2 sample showing the size of SiO_2_ nanoparticles.

EDS point test was carried out for MS2 sample (Figure 2) and the result is shown in Table 2, which shows that the composition of the synthesized composite was probably near the expected range determined by the ASTM AM60 standard and that the casting procedure was carried out correctly. The arrows in Figure 2 indicate Mg_17_Al_12_ intermetallic phase, also detected by the EDS map analysis (Figure 3b), and XRD analysis (Figure 4).

According to Figure 3, the strengthening particles were uniformly distributed in the alloy matrix, which shows that the ultrasonic method is a suitable method for producing composite castings. The uniform distribution of nanoparticles in the matrix improves the physical and mechanical properties of the composite. Figure 4 shows the field-emission scanning electron microscope (FESEM) image of Sample MS2. It is shown that the measured average size of SiO_2_ nanoparticles are was almost 35 nm and that the particles were uniformly distributed in the matrix.

The stabilization of SiO_2_ nanoparticles in molten magnesium depends on different factors, namely external factors such as stirring process, and thermodynamic ones such as reduced van der Waals forces between the nanoparticles in molten magnesium, thermal energy of the nanoparticles, and an energy barrier which prevents SiO_2_ nanoparticle from sintering since they have a significant wettability in molten magnesium [24]. Van der Waals interaction of two sphere SiO_2_ particles in a molten media can be calculated using the following equation at 700 °C, assuming that both particles have radii of *R* [35]:

(1) $$W_{vdw}\left(D\right)=\frac{-{\left(\sqrt{A_{SiO_{2}}}-\sqrt{A_{Mg}}\right)}^{2}}{D}\;\frac{R}{2}$$

where *D* is the distance between two nanoparticles in nm, and $A_{SiO2}$ and $A_{\mathrm{Mg}}$ are the Hamaker constants for the van der Waals interaction. The media surrounding the nanoparticles mainly consists of Mg, and the value of Hamaker constant is 71.6 zJ and 206 zJ for SiO_2_ and molten magnesium, respectively [36]. Since Equation (1) is applicable only when the two particles interact in the melt of Mg with a distance approximately larger than two layers of Mg atom, i.e., ~0.4 nm, the maximum attraction is calculated to be −126.5 zJ. Since the difference between Hamaker constant for Mg and SiO_2_ is relatively large, the van der Waals interaction is high. The thermal energy of nanoparticles for Brownian motion, i.e., $E_{b}$, at 700 °C is calculated to be 13.42 zJ and is not large enough to enable the nanoparticles to break free from their attraction [24]. The interfacial energy barrier is calculated using the following equation:

(2) $$E_{int}=S\left(\sigma_{SiO_{2}}-\sigma_{SiO_{2}-AM60}\right)=S\sigma_{Mg}cos\theta$$

where *S* = *πRD*_0_ is the effective area of two spheres with a diameter of *R* and at *D*_0_ spacing, $\sigma_{\mathrm{SiO}2}$ is the surface energy of SiO_2_, $\sigma_{\mathrm{SiO}2-\mathrm{AM}60}$ is the interfacial energy between SiC and AM60 melt, $\sigma_{\mathrm{AM}60}$ is the surface tension of magnesium melt, and *θ* is the contact angle of magnesium alloy melt on SiO_2_ surface. The contact angle of SiO_2_ in Mg alloy at 700 °C is approximately 80° [37] and the surface energy of Mg alloy at this temperature is 0.433 J m^−2^ [38]. Consequently, using Equation (2) the interfacial energy barrier is 1.64 × 10^3^ zJ. Therefore, the interfacial energy increase prevents the nanoparticles from contacting and sintering. While the thermal energy is not large enough to overcome the van der Waals attraction, the particles may not sinter if they are in contact and hence a uniform distribution of nanoparticles is achieved. The above calculation shows that the particles could be stable without the continuous presence of an external stirring force.

According to the previous reports [33,34], SiO_2_ nanoparticles may undergo chemical reactions with the molten Mg as follows:

4Mg + SiO_2_ = Mg_2_Si + 2MgO

The Gibbs free energy for the above reaction is calculated to be −84 kJ at the superheat temperature for this research, 700 °C, which shows the reaction is thermodynamically favourable. Boruni et al. [33] used amorphous SiO_2_ particles and attributed the improvements in the mechanical properties of their samples solely to the secondary phases produced by Mg-SiO_2_ and Mg-Al reactions in the melt during the casting process, i.e., Mg_2_Si and MgO. However, the possibility of the contribution by the unreacted SiO_2_ particles needs to be taken into account, too, when assessing the mechanical properties of the samples, given the lower melt superheat, and a less severe stirring in this research compared to the work of Brouni et al. In the current work, crystalline SiO_2_ was used as the reinforcement, which is expected to affect the crystal structure of the matrix by acting as a grain refiner.

### 3.2. X-ray Diffraction (XRD) Analysis

**Figure 5:**
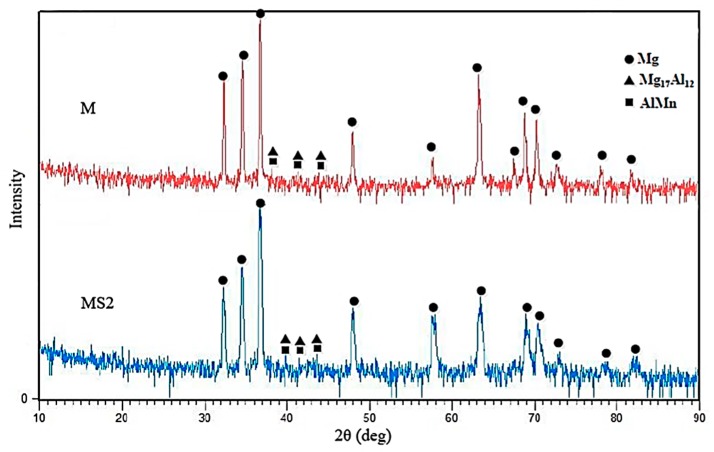
X-ray diffraction pattern of samples of M (AM60) and MS2 (AM60 + 2wt.% SiO_2_).

As Figure 5 shows, the broadening is observable in the width of Mg matrix peaks of MS2 sample, which is probably due to decrease in Mg grain size. The decrease in composite matrix grain size can be attributed to the presence of nanoparticles which are known to act as nucleation sites [8].

The crystallite size of the matrix was calculated using the Scherrer equation expressed as:

(3) $$D=\frac{K\lambda }{B\cos\theta }$$

where *D* is the average crystallite size in nm, *K* is Scherrer constant, *λ* is the X-ray wavelength, *θ* is the Bragg angle and *B* is the full width half maximum (FWHM) of the peaks considered for the calculation. The average crystallite size was calculated to be ~43 nm for sample M while a crystallite size drop of 60% was observed in MS2 sample. Therefore, it can be concluded that crystalline SiO_2_ particles may provide suitable heterogeneous nucleation sites for Mg atoms and hence lead to the reduction in grain size of the matrix. It also suggests that there is a low planar disregistry between SiO_2_ crystalline particles and Mg matrix both with hexagonal crystallographic structures. It should be added that no phase containing Si is identified in the X-ray pattern which can be attributed to low amount of added nanoparticles, 2 wt.% at maximum, which is lower than the resolution of the XRD analysis.

### 3.3. Hardness and Compression Tests

Vickers hardness of samples M and MS2 were 34.8 HV and 51.5 HV, respectively. Generally, hardness and other mechanical properties of metal matrix composites are known to depend on the size and amount of the second strengthening phase. Considering that the particles used in this research were ~35 nm, the dispersion-strengthening mechanism was in effect. In fact, the metallic matrix withstood the subjected load and the particles hindered the motion of the dislocations by mechanisms such as pinning [39].

**Figure 6:**
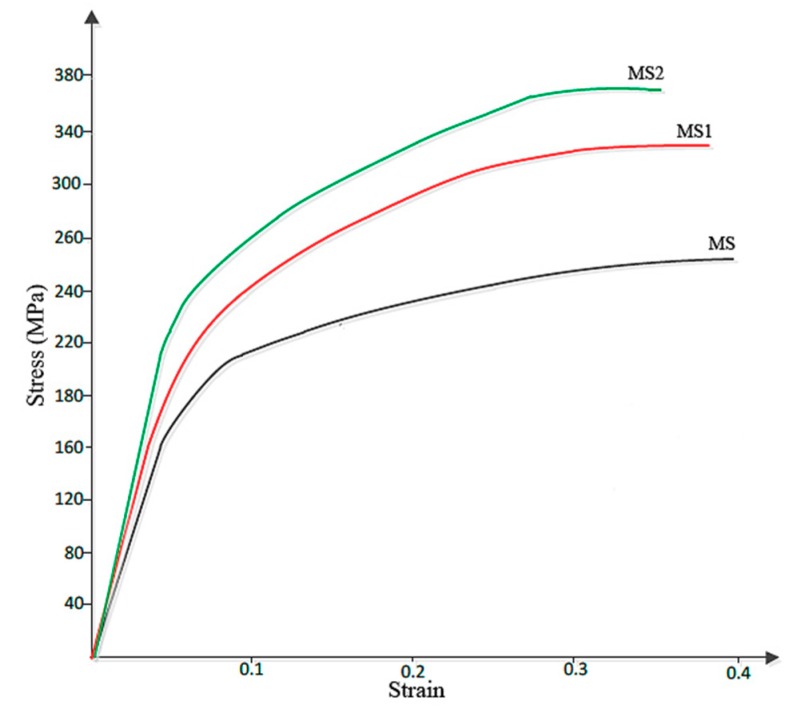
Stress-strain curve obtained from the compression test.

Figure 6 shows the Stress-Strain curve obtained from the compression test. According to this figure, addition of second strengthening phase has led to the increase in the compressive strength and fracture stress of sample MS2, which is almost 44% larger than that of sample M. Fracture strain were 0.41, 0.39 and 0.37, respectively for the samples of M, MS1 and MS2.

Since it is known that HCP metals are mainly yielded due to twining, grain refinement can reduce twinning activity and hence increase the compressive yield strength [5]. On the other hand, grain refinement increases the strength of the crystal according to the Hall–Petch equation [40] expressed as:

(4) $$\sigma =\sigma_{0}+Kd^{-0.5}$$

where *σ*, *σ*_0_, *K* and *d*, are the yield stress, the yield stress of a single crystal, a constant, and the grain size, respectively. The value of *K* generally depends on the number of slip systems and is greater for HCP metals than for FCC and body-centered cubic BCC metals. Then, the strength of Mg with an HCP structure is more sensitive to grain size reduction.

**Figure 7:**
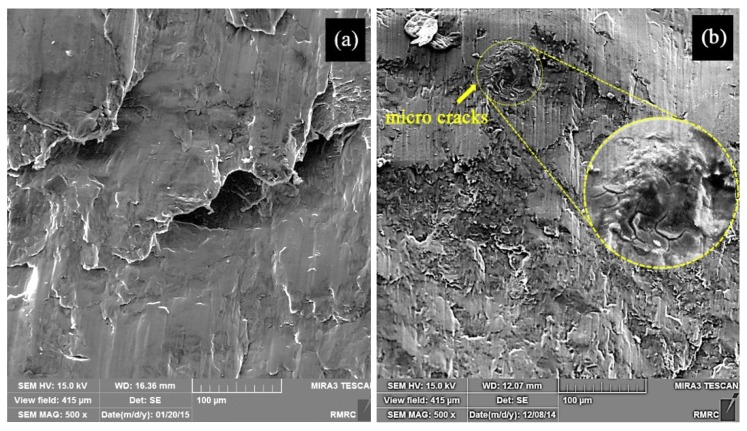
SEM images of the fractured surface of sample (**a**) M, and (**b**) MS2 with the inset showing the crack area with a higher magnification.

Besides the Hall–Petch strengthening mechanism, the significant improvement in hardness and strength of the samples observed in this work can also be attributed to Orowan strengthening mechanism [6], also originated from the introduction of nanoparticles into the matrix. However, it was expected that the decrease in grain size of the ultrafine structured metal matrix would increase the overall ductility to failure of sample MS2 [41], while according to Figure 6, sample M with a larger average grain size showed a larger ductility. This may be attributed to the presence of the nanoparticles which are obstacles against moving dislocations and hence restrict the ability of the sample to undergo plastic deformation. The particles may also act as stress concentration sites and lead to failure of the sample at lower plastic deformation, a finding which is in agreement with that of M. Habibnejad-Korayem et al. [6] who reported that the presence of nanoparticles may have encouraged crack initiation and propagation. Figure 7 shows the SEM images of fractured surface of M and MS2 cylindrical specimens after the compression test.

**Figure 8:**
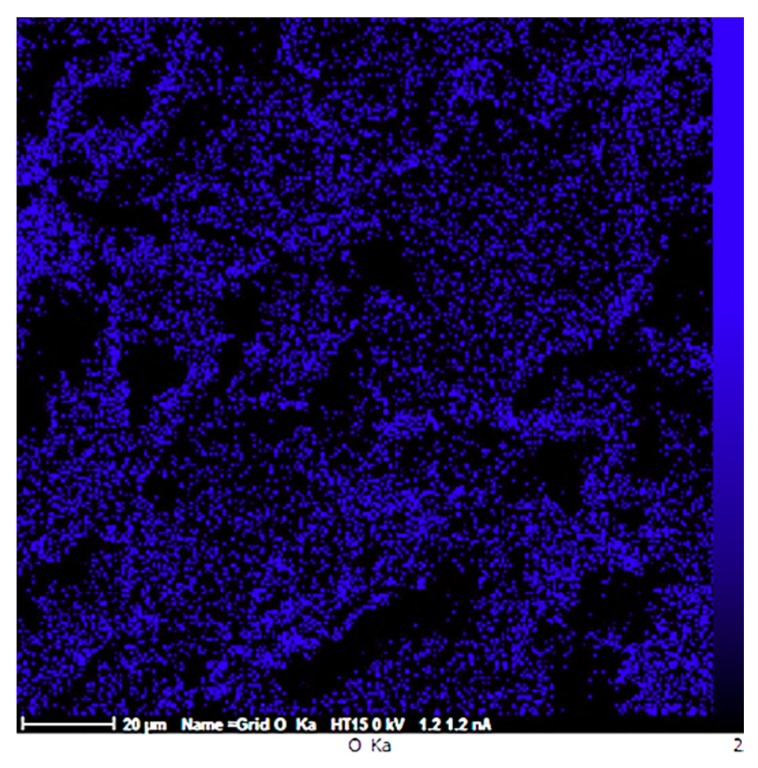
EDS map of sample M showing the distribution of Oxygen.

Micro-cracks leading to the reduced ductility are indicated in Figure 7b. Formation of crack in sample MS2 can be either due to the dislocation pile up and the consequent local stress concentration at the interface between the repulsive prismatic or basal slip dislocations and twins [42], or due to the interaction of the dislocations with SiO_2_-strengthening particles. Since such crack nucleation sites are not visible in sample M (Figure 7a), it can be concluded that the latter mechanism is more probable. However, this is in contrast to the finding of M. Paramsothy et al. [43] who reported improved ductility after the addition of nanoparticles. This difference could largely stem from the different processing method, alloy system and ceramic particle used, which could alter the dominant texture and hence the mechanical behaviour of the composite. Both of the surfaces were featured with shear bands, as expected in compressive fracture, which can be attributed to the heterogeneous plastic deformation at grain boundaries [44]. Large local plastic slip and the consequent local shear bands can be seen between void ligaments, as shown in Figure 7a. Therefore, it can be concluded that the fracture mechanism in sample M was shear fracture with void [45]. Moreover, the laminar appearance of the fracture surface in sample M suggests that formation of double oxide films could have caused the fracture. EDS results obtained prior to compression test on sample M revealed that the sample contained 26.91 wt.% O which could be the result of the turbulence that occurred during the ultrasound assisted-casting process. According to the EDS map of the sample (Figure 8), oxygen is well distributed thoroughly out the sample. Griffiths et al. [46] found folded MgO films in pure Mg casts produced as a result of poor casting conditions.

By contrast, as seen in Figure 7b, fewer voids and a more relatively smooth cleavage surface is seen. Due to the presence of well-dispersed second phase materials (Figure 3) and large amount of grain boundaries, plastic deformation is restricted in sample MS2. In other words, addition of SiO_2_ nanoparticles increased the strength of the sample MS2 and decreased its ductility so that it experienced a brittle fracture [6]. The non-laminar fracture surface of sample MS2 is an indication of better casting conditions, which is in accordance with the results of EDS test carried out prior to compression test revealing that the sample contained less amount of oxygen (Table 1).

## 4. Conclusions

AM60 nanocomposites reinforced with SiO_2_ nanoparticles were synthesized using ultrasound-assisted casting method. The correlation between the reinforcement content and the strengthening efficiency of the SiO_2_ nanoparticles in AM60 magnesium alloy were investigated. The particles were uniformly distributed due to the contribution of an external stirring force and thermodynamic factors. According to the results, SiO_2_ nanoparticles led to reduction in the Mg alloy crystal size which increased the compressive strength and hardness of the sample. Introduction of nanoparticles, however, decreased the ductility of the composites mainly due to the refinement of the HCP Mg grains and hence the reduction in twin activity, and formation of micro-cracks as a result of dislocation interaction with SiO_2_-strengthening particles. The fracture surface showed formation of shear bands in both MS and MS2 samples with crack formation and less plastic deformation in MS2.

## Figures and Tables

**Table 1:** Nominal composition of sample AM60 alloy (wt.%).

Al	Mn	Si	Zn	Fe	Cu	Ni	Others	Mg
5.5–6.5	0.25	0.1	0.22	0.005	0.01	0.002	0.003	Balance

**Table 2:** Chemical composition of the sample MS2.

Element	%W
O	5.0
Mg	86.5
Mn	0.2
Al	6.1
Si	2.2

## References

[1] Mordike B.L., Ebert T. (2001). Magnesium Properties-applications-potential. Mater. Sci. Eng. A.

[2] Kondori B., Mahmudi R. (2014). Effect of Ca additions on the microstructure, thermal stability and mechanical properties of a cast AM60 magnesium alloy. Mater. Sci. Eng. A.

[3] Jinwang Z., Shebin W., Junyuan Z., Jinling Z., Bingshe X. (2009). Effects of Nd on Microstructures and Mechanical Properties of AM60 Magnesium Alloy in Vacuum Melting. Rare Met. Mater. Eng..

[4] Yong H., Li R. (2012). Effects of silicon on mechanical properties of AM60 magnesium alloy. China Foundry.

[5] Tun K., Wong W., Nguyen Q., Gupta M. (2013). Tensile and compressive responses of ceramic and metallic nanoparticle reinforced Mg composites. Materials.

[6] Habibnejad-Korayem M., Mahmudi R., Poole W.J. (2009). Enhanced properties of Mg-based nano-composites reinforced with Al_2_O_3_. Mater. Sci. Eng. A.

[7] Clyne T.W., Withers P.J. (1995). An Introduction to Metal. Matrix Composites.

[8] Ye H.Z., Liu X.Y. (2004). Review of recent studies in magnesium. J. Mater. Sci..

[9] Wang L.G., Zhang B.F., Zhu S.J., Zhang M., Zhang C.X., Guan S.K. (2006). Effects of silicocalcium on microstructure and properties of Mg-6A1-0.5Mn alloy. Trans. Nonferrous Met. Soc. China.

[10] Wang L.G., Zhang B.F., Zhu S.J., Zhang C.X., Guan S.K. (2007). Application of silicocalcium in Mg-6Al-0.5Mn alloy. China Foundry.

[11] Mortensen A., Llorca J. (2010). Metal Matrix Composites. Annu. Rev. Mater. Res..

[12] Saboori A., Moheimani S., Pavese M., Badini C., Fino P. (2017). New nanocomposite materials with improved mechanical strength and tailored coefficient of thermal expansion for electro-packaging applications. Metals.

[13] Zare H., Jahedi M., Toroghinejad M.R., Meratian M., Knezevic M. (2016). Compressive, shear, and fracture behavior of {CNT} reinforced Al matrix composites manufactured by severe plastic deformation. Mater. Des..

[14] Chen J., Bao C., Chen W., Zhang L., Liu J. (2016). Mechanical Properties and Fracture Behavior of Mg-Al/AlN Composites with Different Particle Contents. J. Mater. Sci. Technol..

[15] Aniban N., Pillai R.M., Pai B.C. (2002). An analysis of impeller parameters for aluminium metal matrix composites synthesis. Mater. Des..

[16] Przestacki D., Szymanski P., Wojciechowski S. (2016). Formation of surface layer in metal matrix composite A359/20SiCP during laser assisted turning. Compos. Part. A Appl. Sci. Manuf..

[17] Li S., Su Y., Zhu X., Jin H., Ouyang Q., Zhang D. (2016). Enhanced mechanical behavior and fabrication of silicon carbide particles covered by in-situ carbon nanotube reinforced 6061 aluminum matrix composites. Mater. Des..

[18] Mosallanejad M.H., Shafyei A., Akhavan S. (2016). Simultaneous co-deposition of SiC and CNT into the Ni coating. Can. Metall. Q..

[19] Shi H.L., Wang X.J., Zhang C.L., Li C.D., Ding C., Wu K., Hu X.S. (2016). A Novel Melt Processing for Mg Matrix Composites Reinforced by Multiwalled Carbon Nanotubes. J. Mater. Sci. Technol..

[20] Saboori A., Dadkhah M., Fino P., Pavese M. (2018). An overview of metal matrix nanocomposites reinforced with graphene nanoplatelets; Mechanical, Electrical and Thermophysical properties. Metals.

[21] Tjong S.C. (2007). Novel Nanoparticle-Reinforced Metal Matrix Composites with Enhanced Mechanical Properties. Adv. Eng. Mater..

[22] Esmaily M., Mortazavi N., Svensson J.E., Halvarsson M., Wessén M., Johansson L.G., Jarfors A.E. (2016). A new semi-solid casting technique for fabricating SiC-reinforced Mg alloys matrix composites. Compos. Part B Eng..

[23] Cao G., Konishi H., Li X. (2016). Mechanical Properties and Microstructure of Mg/SiC Nanocomposites Fabricated by Ultrasonic Cavitation Based. J. Manuf. Sci. Eng..

[24] Chen L.Y., Xu J.Q., Choi H., Pozuelo M., Ma X., Bhowmick S., Yang J.M., Mathaudhu S., Li X.C. (2015). Processing and properties of magnesium containing a dense uniform dispersion of nanoparticles. Nature.

[25] Nie K.B., Wang X.J., Hu X.S., Xu L., Wu K., Zheng M.Y. (2011). Microstructure and mechanical properties of SiC nanoparticles reinforced magnesium matrix composites fabricated by ultrasonic vibration. Mater. Sci. Eng. A.

[26] Luo A. (1996). Heterogeneous Nucleation and Grain Refinement in Cast Mg(AZ91)/SiCP Metal Matrix Composites. Can. Metall. Q..

[27] Saboori A., Padovano E., Pavese M., Badini C. (2018). Novel magnesium Elektron21-AlN nanocomposites produced by ultrasound-assisted casting; microstructure, thermal and electrical conductivity. Materials.

[28] Saboori A., Padovano E., Pavese M., Dieringa H., Badini C. (2017). Effect of solution treatment on precipitation behaviors, age hardening response and creep properties of Elektron21 alloy reinforced by AlN nanoparticles. Materials.

[29] Moosbrugger C. (2017). Engineering Properties of Magnesium Alloys.

[30] Do Lee C. (2007). Tensile properties of high-pressure die-cast AM60 and AZ91 magnesium alloys on microporosity variation. J. Mater. Sci..

[31] Easton M., Song W.Q., Abbott T. (2006). A comparison of the deformation of magnesium alloys with aluminium and steel in tension, bending and buckling. Mater. Des..

[32] Medved J., Mrvar P., Voncina M. (2011). Oxidation Resistance of AM60, AM50, AE42 and AZ91 Magnesium Alloys. Magnes. Alloy Corros. Surf. Treat..

[33] Borouni M., Niroumand B., Maleki A. (2017). Synthesis and characterization of an in-situ Magnesium-based cast nano composite via nano-SiO2 additions to the melt. Mater. Technol..

[34] Huang D., Wang Y.L., Wang Y., Cui H.B., Guo X.F. (2011). In situ Mg2Si reinforced Mg alloy synthesized in Mg-SiO2 system. Adv. Mater. Res..

[35] Israelachvili J.N. (2011). Intermolecular and Surface Forces.

[36] Xu J.Q., Chen L.Y., Choi H., Li X.C. (2012). Theoretical study and pathways for nanoparticle capture during solidification of metal melt. J. Phys. Condens. Matter.

[37] Fritze C., Nientit G. (1995). The wettability of oxide ceramics by magnesium alloys. J. Mater. Sci. Lett..

[38] Park S., Yum S., Kum C., Hur B. (2005). Thermophysical properties of Al and Mg alloys for metal foam fabrication. Colloid Surf. A-Physicochem. Eng. Asp..

[39] Garcia I., Fransaer J., Celis J.P. (2001). Electrodeposition and sliding wear resistance of nickel composite coatings containing micron and submicron SiC particles. Surf. Coat. Technol..

[40] Hansen N. (2004). Hall–Petch relation and boundary strengthening. Scr. Mater..

[41] Wang J., Horita Z., Furukawa M., Nemoto M., Tsenev N.K., Valiev R.Z., Ma Y., Langdon T.G. (1993). An investigation of ductility and microstructural evolution in an Al-3 % Mg alloy with submicron grain size. Mater. Res. Soc..

[42] Yoo M.H. (1981). Slip, Twinning, and Fracture in Hexagonal Close-Packed Metals. Metall. Trans. A.

[43] Paramsothy M., Hassan S.F., Srikanth N., Gupta M. (2009). Enhancing tensile/compressive response of magnesium alloy AZ31 by integrating with Al_2_O_3_ nanoparticles. Mater. Sci. Eng. A.

[44] Zhang Z.F., Eckert J., Schultz L. (2003). Difference in compressive and tensile fracture mechanisms of Zr59Cu20Al10Ni8Ti3 bulk metallic glass. Acta Mater..

[45] Li Z., Shi J., Tang A. (2013). Investigation on fracture mechanisms of metals under various stress states. Acta Mech..

[46] Griffiths W.D., Lai N.W. (2007). Double Oxide Film Defects in Cast Magnesium Alloy. Metall. Mater. Trans..

